# Association between acetaminophen administration and outcomes in critically ill patients with gout and hypertension

**DOI:** 10.3389/fphar.2024.1445975

**Published:** 2024-08-12

**Authors:** Xiao-Qing Yi, Bo Xie, Yuan Hu, Tian-Jiao Gong, Min Chen, Xiao-Jiao Cui

**Affiliations:** ^1^ Department of Pharmacy, Personalized Drug Therapy Key Laboratory of Sichuan Province, Sichuan Academy of Medical Sciences and Sichuan Provincial People’s Hospital, School of Medicine, University of Electronic Science and Technology of China, Chengdu, China; ^2^ Department of Cardiology, Chengdu First People’s Hospital, Chengdu, China

**Keywords:** critical illness, mortality, acetaminophen, gout, hypertension

## Abstract

**Background:**

Acetaminophen is a commonly used medication, yet its recommendation for patients with comorbid conditions of gout and hypertension is contradictory, and the impact of its usage on clinical outcomes in real-world practical settings remains uncertain. The aim of this study was to investigate the association between acetaminophen administration and clinical outcomes in critically ill patients with gout and hypertension, utilizing real-world data.

**Methods:**

A retrospective cohort study was conducted based on the MIMIC-IV (Medical Information Mart in Intensive Care-IV) database. Adult critically ill patients with gout and hypertension were included in the analysis. The exposure was acetaminophen use during ICU stay. The primary outcome was in-hospital mortality. The secondary endpoints were frequent hospitalization, 30-day, and 60-day all-cause mortality, and incidence of hypertensive emergencies. Propensity score matching (PSM) was conducted at a 1:1 ratio. Multivariable analyses were used to adjust for confounders.

**Results:**

The pre-matched and propensity score-matched cohorts included 2448 and 1012 patients, respectively. In the PSM analysis, in-hospital mortality was 9.7% (49/506) in the acetaminophen use group and 12.1% (61/506) in the no use group. Acetaminophen use was associated with a decrease in-hospital mortality (hazard ratio [HR], 0.62; 95% CI, 0.41–0.92; *P* = 0.018). In terms of secondary endpoints, after PSM, there was no statistically significant difference for both 30-day and 60-day all-cause mortality reductions in the acetaminophen use group, and HRs were 0.78 (95% CI 0.55–1.11; *P* = 0.175), and 0.75 (95% CI 0.55–1.02; *P* = 0.069), respectively. According to the analysis of dosage and treatment group, the use of APAP within the dosage range of 2–4 g and within 3–5 days of treatment significantly reduced the mortality rate of the entire cohort and PSM cohort, with statistical differences. Subgroup analysis demonstrated that lower in-hospital mortality was consistent across different baselines (age, gender, BMI, liver disease, and renal disease), with no interactions in all subgroups (interaction *p*-values >0.05), thereby affirming the robustness and reliability of the findings.

**Conclusion:**

Acetaminophen use was associated with lower in-hospital mortality in critically ill patients with gout and hypertension. Prospective studies are needed to verify this finding.

## 1 Introduction

Gout is the most common inflammatory arthritis in adults, affecting approximately 41.2 million adults worldwide (Danve et al., 2021). It is precipitated by sustained hyperuricemia, leading to the deposition of monosodium urate crystals in joints, tendons, and other tissues. This accumulation triggers recurrent bouts of acute inflammation, known as gout flares (Dehlin et al., 2020). Hypertension and cardiovascular diseases are among the most common comorbidities of gout and hyperuricaemia (Kuwabara et al., 2017; Sandoval-Plata et al., 2021), which are associated with increased morbidity and mortality risk (Singh and Gaffo, 2020). Hypertension predisposes to gout by reducing renal urate excretion due to glomerular arteriolar damage and glomerulosclerosis, and studies have shown that hypertension is an independent risk factor for gout (Kuo et al., 2016; Evans et al., 2018). A survey found that among the 3.9% of surveyed individuals with gout, 74% had hypertension (Zhu et al., 2012).

Acetaminophen (also known as paracetamol) is widely used worldwide, it has a spectrum of action similar to that of NSAIDs and resembles particularly the COX-2 selective inhibitors (Graham et al., 2013). Due to its weak anti-inflammatory activity (Graham et al., 2013), acetaminophen is usually not recommended as the main treatment for gout (Richette et al., 2017; FitzGerald et al., 2020; Richette et al., 2020; Neilson et al., 2022), but some gout patients may need it to relieve pain or reduce fever when taking other gout medications. Acetaminophen is often preferred because it is generally well tolerated and considered safer than other analgesics (Graham et al., 2013; Alchin et al., 2022), especially previous researches have shown that NSAIDs increase cardiovascular risk (McGettigan and Henry, 2011; Coxib and traditional NSAID Trialists’ CNT Collaboration et al., 2013). The guideline of the American Heart Association recommends the use of acetaminophen in hypertension patients with pain (Whelton et al., 2018). However, and recent research reports that acetaminophen increases hypertension (Dawson et al., 2013; Benitez-Camps et al., 2018; MacIntyre et al., 2022), and increases the risk of cardiovascular adverse consequences (Zeng et al., 2022). Meanwhile, another multicenter cohort study targeting the ICU population showed that half of patients receiving intravenous injection of acetaminophen experienced hypotension, and up to one-third of observed episodes required therapeutic intervention (Cantais et al., 2016). There has been no further research evaluating the impact of acetaminophen on the mortality of critically ill patients with gout and hypertension. The aim of this study was to investigate the actual use of acetaminophen in gout patients with hypertension admitted to ICU, and further evaluates the impact of acetaminophen use on mortality in this patient population.

## 2 Methods

### 2.1 Data source

This study was a retrospective cohort study based on the MIMIC-IV database (Johnson and Bulgarelli, 2024). MIMIC-IV is a publicly available database sourced from the electronic health record of the Beth Israel Deaconess Medical Center and record dataset covering a decade of admissions between 2008 and 2019. The Institutional Review Board at the Beth Israel Deaconess Medical Center granted a waiver of informed consent and approved the sharing of the research resource. Author Yi passed the online training courses and exams (certification number: 59888607). MIMIC-IV establishes a modular organization of the constituent data allowing linking of the database to external departments and distinct modalities of data which allowed us to explore the availability of out-of-hospital mortality.

### 2.2 Study population

Patients whose diagnostic description included both “Gout” and “Hypertension” were enrolled in the study. A total of 16086 hospitalization records of patients with Gout and hypertension were collected in the MIMIC-IV database. We excluded patients who were under 18 years of age, were not admitted to the ICU, or had an ICU stay of less than 24 h. If the patient had multiple ICU admissions, only the first ICU admission was analysed.

### 2.3 Exposure and outcomes

The exposure of interest was the use of acetaminophen during the ICU stay, without any restrictions. Acetaminophen use was extracted from the prescriptions table. The primary outcome was in-hospital mortality. Secondary outcomes included 30-day all-cause mortality, 60-day all-cause mortality, and length of stay at the hospital (LOS), and incidence of hypertensive emergencies. According to the guidelines of the ESC Hypertension Committee (van den Born et al., 2019), we consider the occurrence of systolic blood pressure >200 mmHg or diastolic blood pressure >120 mmHg during ICU hospitalization as hypertensive emergencies.

### 2.4 Data extraction

Baseline variables for the first 24 h of ICU admission were gathered from the MIMIC-IV database. Patient characteristics, including age, gender, race, and BMI, were collected. We extracted information on concomitant medications, including β-blockers, angiotensin-converting enzyme inhibitors (ACEI), angiotensin receptor blockers (ARB), calcium channel blockers (CCB), diuretics, colchicine, NSAIDs, glucocorticoids, antibiotics and Vasoactive drugs. We extracted information on comorbidities, such as myocardial infarction, congestive heart failure, peripheral vascular disease, cerebrovascular disease, chronic pulmonary disease, rheumatic disease, peptic ulcer disease, diabetes, renal disease, malignant cancer, and liver disease. The following variables were extracted, and for variables with multiple measured values, calculate the average value within 24 h to reduce the impact of their variation: systolic blood pressure (SBP), diastolic blood pressure (DBP), heart rate, respiratory rate, temperature, pulse oxygen saturation (SpO2), sequential organ failure assessment (SOFA), WBC, platelet, ALT, AST, serum creatinine, and serum glucose.

### 2.5 Statistical analysis

The study cohort was divided into two groups: those who received acetaminophen (APAP group) and those who did not (no-APAP group). A patient with a serum glucose value of 143054 and 171 patients with a BMI greater than 100 or less than 10 were considered as outliers. Multiple imputation was used to estimate missing values for each variable in our study (Sterne et al., 2009; Mackinnon, 2010). Mean (standard deviation [SD]) and median (interquartile range [IQR]) should be used for description of normally and non-normally distributed data, respectively (Habibzadeh, 2017). Categorical variables were presented as counts and percentages, which compared by Pearson’s chi-squared test. We used propensity score matching (PSM) to adjust variables according to the recommendations in the literature (Lonjon et al., 2017). The matching was constructed based on a 1:1 ratio using the nearest neighbour method with a calliper width of 0.05 without replacement. The balance of variables between the groups before and after matching was assessed using standardised mean difference (SMD), with a value of less than 0.10 indicating balance (Austin, 2009). Univariate analyses were used to explore the variables associated with death. Statistically significant variables (*p* < 0.05) were brought into multivariate analysis as covariates for further analysis. Multivariate Cox regression analyses with results expressed as hazard ratios (HR) with 95% CI were used to assess the relationship between acetaminophen and mortality. Three multivariate models were used. Cumulative incidence of in-hospital mortality was analysed with the Kaplan-Meier method and evaluated by the log-rank test. Subgroup analyses were performed for the PSM cohort based on age, sex, race, BMI, liver disease, and kidney disease.

All the analyses were performed with the statistical software packages R (http://www.R-project.org, The R Foundation) and Free Statistics software versions 1.9.

## 3 Results

### 3.1 Patient selection

**FIGURE 1:**
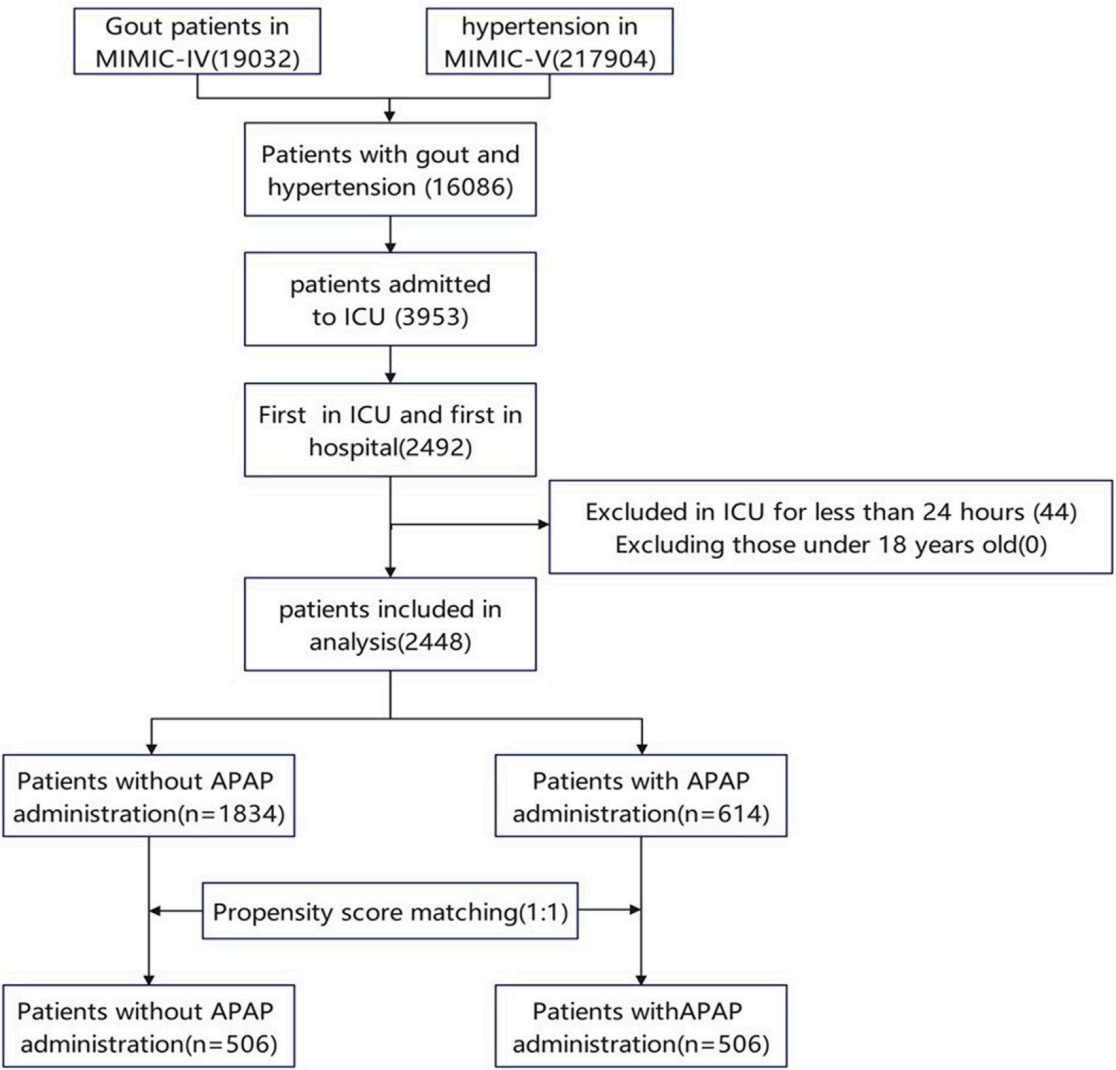
Flow chart of patient selection. MIMIC IV, Multiparameter Intelligent Monitoring in Intensive Care Database IV; ICU, intensive care unit; PSM, propensity-score matching.

The initial search from the MIMIC-IV database identified 16086 patients with gout and hypertension. 3953 cases were admitted to the ICU. The final study population consisted of 2448 patients, with 614 using acetaminophen and 1834 not using it. This population retained their first stay in the ICU, excluding patients under 18 years old and those who had been in the ICU for less than 24 h (Figure 1).

### 3.2 Baseline characteristics

Table 1 shows the baseline characteristics before and after matching. In the entire cohort, patients who received acetaminophen tended to be younger (see additional file: Supplementary Table S2). Patients who received treatment with acetaminophen upon admission had a higher WBC, a lower platelet, and a lower serum glucose value (see additional file: Supplementary Table S2). In terms of vital signs, compared to the no-APAP group, the APAP group had higher body temperature and SOFA scores, while SBP and DBP were lower. Patients receiving acetaminophen treatment have fewer comorbidities but more concomitant medications, especially the proportion of antibiotics and vasoactive drugs used, which was significantly higher in the APAP group. (see additional file: Supplementary Table S2). In general, patients in the APAP group were likely more severe.

**TABLE 1:** Baseline characteristics before and after propensity score matching.

Variables	Entire cohort (n = 2448)		SMD	PSM cohort (n = 1012)		SMD
	No-APAP (n = 1834)	APAP (n = 614)		No-APAP (n = 506)	APAP (n = 506)	
Demographics						
Age, year	73.5 ± 12.1	71.9 ± 10.6	0.134	72.8 ± 11.4	72.3 ± 11.1	0.044
Gender, Male, n (%)	1326 (72.3)	491 (80)	0.181	389 (76.9)	390 (77.1)	0.005
Race, white, n (%)	1275 (69.5)	413 (67.3)	0.049	362 (71.5)	341 (67.4)	0.09
BMI, kg/m2	30.9 ± 7.8	31.1 ± 7.1	0.022	30.8 ± 7.3	31.1 ± 7.1	0.047
Laboratory Examination						
WBC, K/μL	10.1 (7.6, 13.6)	12.6 (9.6, 15.9)	0.184	11.1 (8.1, 14.5)	12.6 (9.5, 15.8)	0.012
Platelet, K/µL	185.1 (141.4, 242.0)	156.3 (126.3, 204.2)	0.338	167.5 (125.0, 212.6)	159.5 (129.1, 212.8)	0.012
ALT, U/L	25.0 (16.0, 49.0)	23.8 (15.0, 46.4)	0.027	25.0 (16.0, 50.5)	25.0 (16.0, 49.0)	0.08
AST, U/L	32.5 (21.0, 60.0)	35.0 (22.0, 61.9)	0.044	35.0 (21.0, 64.0)	35.0 (22.0, 63.6)	0.054
Serum creatinine, mg/dL	1.4 (1.0, 2.3)	1.2 (0.9, 1.7)	0.23	1.3 (1.0, 2.0)	1.3 (1.0, 1.8)	0.054
Serum glucose, mg/dL	130.5 (108.5, 162.9)	128.0 (109.0, 152.5)	0.113	129.0 (108.1, 158.9)	129.2 (110.2, 153.4)	0.012
Concomitant Medication						
βblocker, n (%)	1443 (78.7)	557 (90.7)	0.339	460 (90.9)	453 (89.5)	0.047
ACEI, n (%)	561 (30.6)	167 (27.2)	0.075	154 (30.4)	141 (27.9)	0.057
ARB, n (%)	242 (13.2)	76 (12.4)	0.024	66 (13)	64 (12.6)	0.012
CCB, n (%)	488 (26.6)	222 (36.2)	0.207	172 (34)	168 (33.2)	0.017
Diuretics, n (%)	1211 (66)	508 (82.7)	0.39	400 (79.1)	403 (79.6)	0.015
Colchicine, n (%)	317 (17.3)	75 (12.2)	0.143	65 (12.8)	70 (13.8)	0.029
NSAIDs, n (%)	84 (4.6)	29 (4.7)	0.007	23 (4.5)	24 (4.7)	0.009
Glucocorticoids, n (%)	295 (16.1)	85 (13.8)	0.063	88 (17.4)	77 (15.2)	0.059
antibiotic	1075 (58.6)	549 (89.4)	0.75	438 (86.6)	441 (87.2)	0.018
Vasoactive drugs	513 (28)	398 (64.8)	0.795	294 (58.1)	295 (58.3)	0.004
Comorbidities						
Myocardial infarct, n (%)	441 (24)	154 (25.1)	0.024	127 (25.1)	127 (25.1)	<0.001
Congestive heart failure, n (%)	862 (47)	226 (36.8)	0.208	204 (40.3)	199 (39.3)	0.02
Peripheral vascular disease, n (%)	313 (17.1)	106 (17.3)	0.005	82 (16.2)	86 (17)	0.021
Cerebrovascular disease, n (%)	282 (15.4)	93 (15.1)	0.006	88 (17.4)	82 (16.2)	0.032
Chronic pulmonary disease, n (%)	500 (27.3)	151 (24.6)	0.061	115 (22.7)	128 (25.3)	0.06
Rheumatic disease, n (%)	82 (4.5)	22 (3.6)	0.045	21 (4.2)	19 (3.8)	0.02
Peptic ulcer disease, n (%)	64 (3.5)	9 (1.5)	0.13	7 (1.4)	8 (1.6)	0.016
Diabetes, n (%)	799 (43.6)	233 (37.9)	0.115	202 (39.9)	202 (39.9)	<0.001
Renal disease, n (%)	973 (53.1)	244 (39.7)	0.269	222 (43.9)	219 (43.3)	0.012
Liver disease, n (%)	175 (9.5)	37 (6)	0.132	32 (6.3)	36 (7.1)	0.032
Vital signs						
Heart rate	81.4 ± 15.2	82.4 ± 13.4	0.069	82.4 ± 14.6	82.6 ± 13.9	0.019
SBP, mmHg	121.3 ± 17.1	116.0 ± 13.9	0.335	117.3 ± 15.5	116.8 ± 14.4	0.036
DBP, mmHg	62.4 ± 11.3	59.2 ± 9.6	0.314	59.3 ± 9.6	59.6 ± 9.9	0.026
Respiratory rate	19.3 ± 3.6	18.7 ± 3.1	0.186	18.7 ± 3.5	18.9 ± 3.2	0.066
Temperature (°C)	36.7 ± 0.5	36.8 ± 0.5	0.176	36.8 ± 0.5	36.8 ± 0.5	0.005
Spo2	96.6 ± 2.2	97.2 ± 1.9	0.291	97.2 ± 1.7	97.1 ± 2.0	0.059
Scoring systems						
SOFA	4.0 (2.0, 6.0)	6.0 (4.0, 8.0)	0.458	6.0 (4.0, 8.0)	6.0 (4.0, 8.0)	0.029
Outcome						
In-hospital mortality, n (%)	160 (8.7)	55 (9)		61 (12.1)	49 (9.7)	
LOS hospital, days	7.0 (4.3, 11.4)	8.7 (6.1, 13.9)		8.6 (5.3, 13.0)	8.7 (6.1, 14.2)	
30-day mortality, n (%)	229 (12.5)	66 (10.7)		72 (14.2)	63 (12.5)	
60-day mortality, (%)	312 (17)	84 (13.7)		98 (19.4)	77 (15.2)	
Hypertensive emergencie, (%)	309 (16.8)	111 (18.1)		99 (19.6)	96 (19)	

APAP, acetaminophen; SMD, standardised mean difference.

After PSM, 1012 patients were enrolled, with 506 patients receiving acetaminophen treatment matched to 506 patients who did not receive acetaminophen treatment. The SMD of all variables was<0.1, indicating a similar distribution of baseline variables between the two groups (Table 1).

### 3.3 Relationship between acetaminophen and mortality rate

The overall in-hospital mortality was 8.8% (215/2448). The in-hospital mortality of the APAP group was 9% (55/614), compared with 8.7% (160/1834) for the no-APAP group in Table 1.

In the multivariate Cox regression analysis, we adjusted three models, which included covariates that showed significant differences (P < 0.05) in the univariate analysis. Compared with patients who were not administered APAP, patients who received APAP during ICU stay were associated with a 33% decrease in the risk of in-hospital mortality in the unadjusted model (HR, 0.67; 95% CI, 0.46–0.98; P = 0.041) (Table2). After adjusting for confounding factors, the HR for APAP administration in the multivariate analysis was 0.62 (95% CI, 0.45–0.86; *p* = 0.005) (Table3). The results of the IPTW (HR: 0.67%, 95% CI 0.5–0.91, *p* = 0.048) and PSM (HR: 0.62%, 95% CI 0.41–0.92, *p* = 0.018) models demonstrated a significant beneficial effect of APAP use on in-hospital mortality among ICU patients (see additional file: Supplementary Table S3). At the same time, there was a significant difference in the reduction of hypertensive emergencies in the acetaminophen group (HR: 0.68%, 95% CI 0.5–0.92; *p* = 0.013) (Table 3). In terms of secondary endpoints, after PSM, in the multivariate Cox regression analysis, there was no statistically significant difference for both 30-day and 60-day all-cause mortality reductions in the acetaminophen use group, and HRs were 0.78 (95% CI 0.55–1.11; *p* = 0.175) and 0.75 (95% CI 0.55–1.02; *p* = 0.069), respectively (Table 3). Another secondary endpoint showed a correlation between acetaminophen use and prolonged hospital stay in the multivariate analysis. (see additional file: Supplementary Table S4).

**TABLE 2:** Analysis of the impact of potential confounders of in-hospital mortality association.

	HR (95% CI)	P (Wald’s test)
Age	1.04 (1.02,1.06)	<0.001
Male	0.94 (0.62,1.45)	0.792
White	0.7 (0.48,1.03)	0.068
BMI	0.97 (0.94,0.99)	0.015
WBC	1.01 (1,1.02)	0.001
Platelet	0.9994 (0.9972,1.0015)	0.557
ALT	1.0005 (0.9999,1.0012)	0.131
AST	1.0003 (1,1.0006)	0.091
Serum creatinine	1.14 (1.04,1.25)	0.003
Serum glucose	1.0029 (0.9998,1.0059)	0.063
βblocker	0.35 (0.22,0.56)	<0.001
ACEI	0.53 (0.32,0.87)	0.013
ARB	0.05 (0.01,0.34)	0.002
CCB	0.52 (0.33,0.79)	0.003
Diuretics	0.61 (0.38,0.96)	0.034
Colchicine	0.42 (0.2,0.87)	0.019
NSAIDs	0.43 (0.11,1.75)	0.238
Glucocorticoids	1.02 (0.65,1.59)	0.936
antibiotic	0.95 (0.53,1.69)	0.852
Vasoactive drugs	1.64 (1.09,2.48)	0.018
Myocardial infarct	1.15 (0.76,1.74)	0.513
Congestive heart failure	1.53 (1.04,2.24)	0.031
Peripheral vascular disease	0.73 (0.42,1.29)	0.28
Cerebrovascular disease	1.23 (0.79,1.91)	0.357
Chronic pulmonary disease	0.54 (0.33,0.9)	0.018
Rheumatic disease	1.04 (0.42,2.55)	0.937
Peptic ulcer disease	0.66 (0.16,2.7)	0.568
Diabetes	0.8 (0.55,1.19)	0.272
Renal disease	1.36 (0.94,1.99)	0.106
Liver disease	1.68 (0.99,2.84)	0.053
Heart rate	1.01 (1,1.02)	0.078
SBP	0.97 (0.96,0.98)	<0.001
DBP	0.98 (0.96,1)	0.021
Respiratory rate	1.1 (1.04,1.15)	<0.001
Temperature	0.92 (0.64,1.34)	0.668
Spo2	0.95 (0.88,1.03)	0.245
SOFA	1.14 (1.08,1.19)	<0.001
APAP	0.67 (0.46,0.98)	0.041

**TABLE 3:** Multivariable Cox regression analysis for mortality.

Categories	Model I		Model II		Model III	
	HR (95% CI)	*p*-value	HR (95% CI)	*p*-value	HR (95% CI)	*p*-value
primary outcome						
In-hospital mortality						
Entire cohort (APAP)	0.73 (0.54–0.99)	0.044	0.74 (0.54–1.01)	0.062	0.62 (0.45–0.86)	0.005
PSM cohort (APAP)	0.67 (0.46–0.98)	0.041	0.66 (0.45–0.96)	0.03	0.62 (0.41–0.92)	0.018
second outcome						
30-day mortality						
Entire cohort (APAP)	0.85 (0.65–1.12)	0.258	0.96 (0.73–1.27)	0.77	0.78 (0.58–1.05)	0.103
PSM cohort (APAP)	0.87 (0.62–1.22)	0.407	0.9 (0.64–1.26)	0.542	0.78 (0.55–1.11)	0.175
60-day mortality						
Entire cohort (APAP)	0.79 (0.62–1.01)	0.06	0.88 (0.69–1.13)	0.317	0.72 (0.55–0.94)	0.014
PSM cohort (APAP)	0.77 (0.57–1.04)	0.093	0.81 (0.6–1.09)	0.159	0.75 (0.55–1.02)	0.069
Hypertensive emergencies						
Entire cohort (APAP)	0.73 (0.58–0.9)	0.004	0.72 (0.58–0.9)	0.004	0.81 (0.64–1.03)	0.081
PSM cohort (APAP)	0.76 (0.57–1.01)	0.058	0.71 (0.54–0.95)	0.022	0.68 (0.5–0.92)	0.013

Model I: did not adjust any variables.

Model II: adjusted for age, gender, race, BMI.

Model III: adjusted for model II, covariates, βblocker, ACEI, ARB, CCB, diuretics, colchicine, vasoactive drugs, congestive heart failure, chronic pulmonary disease, SBP, DBP, respiratory rate; WBC, serum creatinine, SOFA.

**FIGURE 2:**
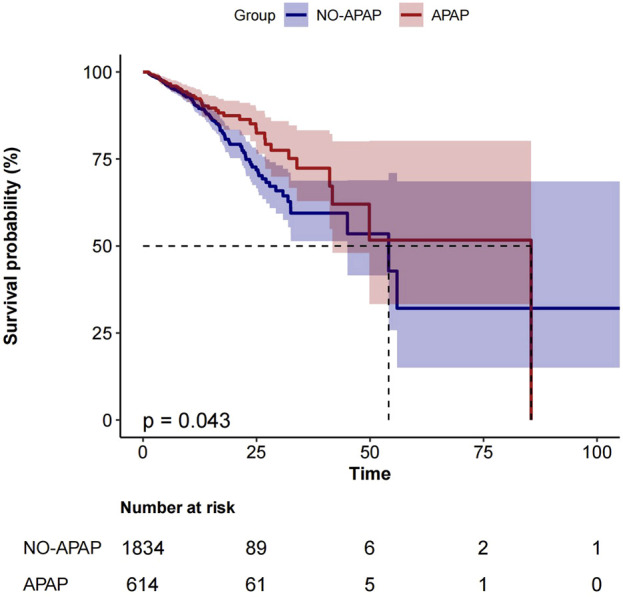
Kaplan–Meier curve for in-hospital mortality according to APAP use in the entire cohort.

Figure 2 shows the Kaplan-Meier curve for in-hospital mortality according to acetaminophen use in the entire cohort.

We analyzed the effect of APAP dosage on in-hospital mortality, and divided the daily dose into three groups: ≤2 g, 2 g ∼ 4 g and >4 g.The results showed that in the entire cohort (HR, 0.47; 95% CI, 0.26–0.83; *p* = 0.01) and PSM cohort (HR, 0.47; 95% CI, 0.25–0.9; *p* = 0.023), the use of 2–4 g doses of acetaminophen can reduce in-hospital mortality, with statistical differences (Table 4). In addition, we analyzed the impact of APAP treatment duration on in-hospital mortality, dividing treatment duration into three groups: ≤3 days, 3–5 days, and>5 days. In the entire cohort (HR, 0.25; 95% CI, 0.09–0.7; *p* = 0.009) and the post PSM cohort (HR, 0.17; 95% CI: 0.04–0.75; *p* = 0.019), treatment with acetaminophen for 3–5 days reduced in-hospital mortality with statistical differences (Table 5).

**TABLE 4:** Multivariable Cox regression analysis of daily dose on in-hospital mortality rate.

Daily dose(g)	n.total	n.event_%	crude.HR (95%CI)	crude.*p*-value	adj.HR (95%CI)	adj.*p*-value
Entire cohort (APAP)						
Not used	1834	160 (8.7)	1(Ref)		1(Ref)	
≤2 g	427	41 (9.6)	0.86 (0.61–1.22)	0.403	0.7 (0.49–1.01)	0.058
2∼4 g	182	14 (7.7)	0.52 (0.3–0.89)	0.018	0.47 (0.26–0.83)	0.01
>4 g	5	0 (0)	0 (0∼Inf)	0.99	0 (0∼Inf)	0.994
P for trend	2448	215 (8.8)	0.75 (0.61–0.94)	0.012	0.69 (0.54–0.87)	0.002
PSM cohort (APAP)						
Not used	506	61 (12.1)	1(Ref)		1(Ref)	
≤2 g	355	37 (10.4)	0.78 (0.52–1.18)	0.24	0.7 (0.45–1.08)	0.107
2∼4 g	146	12 (8.2)	0.49 (0.26–0.92)	0.025	0.47 (0.25–0.9)	0.023
>4 g	5	0 (0)	0 (0∼Inf)	0.993	0 (0∼Inf)	0.994
P for trend	1012	110 (10.9)	0.71 (0.54–0.93)	0.012	0.68 (0.51–0.91)	0.009

Adj: adjusted for age, gender, race, BMI, β-blocker, ACEI, ARB, CCB, diuretics, colchicine, vasoactive drugs, congestive heart failure, chronic pulmonary disease, SBP, DBP, respiratory rate; WBC, serum creatinine, SOFA.

**TABLE 5:** Multivariable Cox regression analysis of in-hospital mortality rate using APAP treatment course.

Course of treatment (day)	n.total	n.Event_%	crude.HR (95%CI)	crude.*p*-value	adj.HR (95%CI)	adj.*p*-value
Entire cohort (APAP)						
Not used	1834	160 (8.7)	1(Ref)		1(Ref)	
≤3d	538	47 (8.7)	0.84 (0.6–1.16)	0.279	0.74 (0.53–1.04)	0.088
3∼5d	49	4 (8.2)	0.4 (0.15–1.08)	0.072	0.25 (0.09–0.7)	0.009
>5d	27	4 (14.8)	0.42 (0.15–1.13)	0.087	0.33 (0.12–0.93)	0.035
P for trend	2448	215 (8.8)	0.75 (0.61–0.94)	0.011	0.66 (0.53–0.84)	0.001
PSM cohort (APAP)						
Not used	506	61 (12.1)	1(Ref)		1(Ref)	
≤3d	453	44 (9.7)	0.76 (0.52–1.12)	0.169	0.71 (0.47–1.07)	0.101
3∼5d	35	2 (5.7)	0.24 (0.06–0.97)	0.045	0.17 (0.04–0.75)	0.019
>5d	18	3 (16.7)	0.44 (0.14–1.42)	0.17	0.39 (0.11–1.34)	0.135
P for trend	1012	110 (10.9)	0.7 (0.53–0.93)	0.013	0.65 (0.48–0.88)	0.005

Adj: adjusted for age, gender, race, BMI, β-blocker, ACEI, ARB, CCB, diuretics, colchicine, vasoactive drugs, congestive heart failure, chronic pulmonary disease, SBP, DBP, respiratory rate; WBC, serum creatinine, SOFA.

### 3.4 Subgroup analyses

**FIGURE 3:**
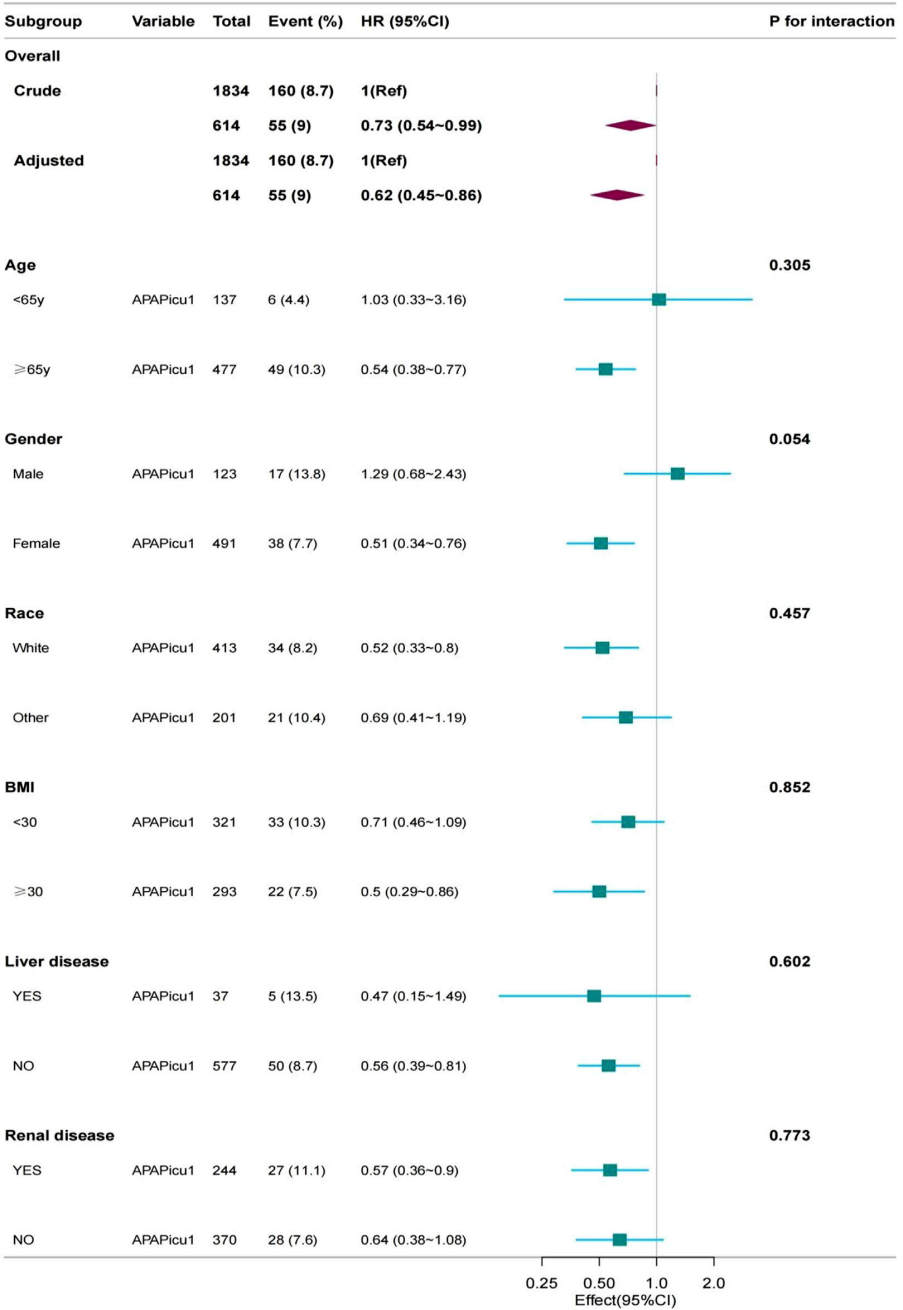
Subgroup analyses for in-hospital mortality. The multivariable Cox proportional hazards model was adjusted for βblocker, ACEI, ARB, CCB, diuretics, colchicine, vasoactive drugs, congestive heart failure, chronic pulmonary disease, SBP, DBP, respiratory rate, WBC, serum creatinine, SOFA. CI, confidence interval; HR, hazard ratio; APAPicu1, acetaminophen was used in the ICU.

A subgroup analysis was performed on the PSM cohort. We used age (<65, ≥65 years), gender (female, male), race (white, other, BMI (<30, ≥30), liver disease, and renal disease as stratification variables to observe the effect values and generate a forest plot of data (Figure 3).We did not observe any significant interactions in all subgroups (*p*-values of interactions were>0.05). Subgroup analysis showed that the relationship remained robust and reliable.

## 4 Discussion

Our study shows that acetaminophen administration is associated with lower in-hospital mortality in ICU patients with gout and hypertension. Even after adjusting for risk factors and applying Cox regression, these results still maintain their strength. The association between acetaminophen use and mortality was broadly consistent across subgroups. The use of acetaminophen is associated with prolonged hospital stay, possibly due to the fact that patients in the acetaminophen group have a relatively more severe initial condition and may have longer hospital stays, resulting in a higher probability of using acetaminophen. The use of acetaminophen reduced all-cause mortality by 30 and 60 days, but there was no significant difference, possibly because 75% of patients in both groups had a total hospital stay within 15 days. Therefore, we believe that the use of acetaminophen in the acute phase has greater benefits. Currently, there are no studies on the impact of acetaminophen use on mortality in ICU populations with gout and hypertension.

There is limited research on acetaminophen in adult ICU patients, and previous studies mainly focused on sepsis patients (Selladurai et al., 2011; Sun et al., 2024). In our study, patients with gout and hypertension who used acetaminophen during ICU stays were included. The usage rate of acetaminophen in this study was 33.5%, which is lower than previous studies (approximately 58%–70%) (Selladurai et al., 2011; Niven et al., 2013), and is related to differences in the study population, as most sepsis patients have fever and higher usage rates. It is worth noting that the use of colchicine and glucocorticoids in the combined medication information of the two groups in the entire queue is significantly higher than that of NSAIDs, and the use of these two types of drugs in the APAP group is relatively lower than that in the no-APAP group. It may be generally believed that acetaminophen is safer, and doctors are more inclined to choose this drug when combined with hypertension. In addition, throughout the entire queue, during the ICU period, the proportion of antibacterial and vasoactive drugs used in the APAP group was significantly higher than that in the non APAP group, possibly due to the poorer general condition of patients in the APAP group. In this study, we corrected for bias caused by factors such as the above and analyzed in-hospital mortality after PSM.

The analgesic and antipyretic effects of acetaminophen are well-established, but the underlying mechanisms of its potential protective effect on mortality are not fully understood. The analgesic and antipyretic effects of acetaminophen are due to its selective action on cyclooxygenase in the central and peripheral nervous system, which inhibits the conversion of arachidonic acid into prostaglandins, thromboxanes, and prostacyclins (Warner and Mitchell, 2004). Acetaminophen reinforces descending inhibitory pain pathways (Pickering et al., 2008), and a study has confirmed that the analgesic mechanism of acetaminophen may involve the serotonin system (Pickering et al., 2006), while the anti-inflammatory activity is very weak, and the guidelines do not specify the use of acetaminophen for gout patients. Glucocorticoid is the first-line treatment recommended by the guidelines, but long-term high-dose use of glucocorticoid has many side effects (such as Cushing’s syndrome, osteoporosis, diabetes and hypertension). Some studies suggest that moderate doses of oral prednisolone plus oral acetaminophen should be used as the first-line treatment for acute gout (Man et al., 2007). Compared with NSAIDs, acetaminophen has higher overall gastrointestinal safety (Hinz et al., 2008). Our study corrected the influencing factors of patients with concomitant peptic ulcer disease.

Acetaminophen is an effective and specific blood protein reducing agent that can block cell-free hemoglobin induced lipid and other substrate oxidation, reducing oxidative damage (Boutaud et al., 2010; Boutaud and Roberts, 2011). Cell-free hemoglobin is an important new predictive factor for survival in patients with severe sepsis and ARDS (40, 41), as it increases inflammation, pulmonary edema, and microvascular permeability (Adamzik et al., 2012; Shaver et al., 2017; Kerchberger et al., 2019; Meegan et al., 2020), and its elevated plasma concentration has been also identified as an independent risk factor for AKI(44) (Graw et al., 2022). This may be one of the reasons for the reduced in-hospital mortality rate in critically ill patients with gout and hypertension in this study. Because after PSM, nearly 90% of the two groups of patients were treated with antibiotics and nearly 60% were treated with vasoactive drugs, there is a higher possibility of sepsis or septic shock in the ICU. The use of APAP weakened the oxidative damage of acellular hemoglobin, thus reducing hospitalization mortality. In addition, acetaminophen can inhibit other peroxidase including myeloperoxidase, reduce the formation of halogenated oxidants (such as hypochlorite and hypobromous acid), and may slow down the development of atherosclerosis and other diseases (Graham et al., 2013).

COXs are highly expressed in renal structures related to volume and pressure control, including cortical and medullary collecting ducts, mesangial cells and dense plaques, as well as medullary interstitial cells in the glomerular vascular system (Francois and Coffman, 2004; Hao and Breyer, 2008). NSAIDs induce hypertension, sodium retention and edema by inhibiting COXs in the kidney (Grosser et al., 2017). There is a similar mechanism for acetaminophen, although its inhibitory activity is weaker compared to NSAIDs. Research reports indicate that acetaminophen, like most other NSAIDs, increases blood pressure. A retrospective observational study reported that among 2754 hypertensive patients treated with acetaminophen, there was a slight increase in blood pressure during treatment with acetaminophen, while the antihypertensive treatment regimen remained unchanged (Dawson et al., 2013). A double-blind, crossover study targeting hypertensive patients with osteoarthritis showed that both acetaminophen and naproxen can have varying degrees of impact on the treatment of hypertension with ramipril, valsartan, or aligilem (Gualtierotti et al., 2013). A double-blind, placebo-controlled, crossover study published in 2022 showed that administering 4 g of Acetaminophen to 110 hypertensive patients for 14 consecutive days increased systolic blood pressure by 4.7 mmHg compared to placebo (MacIntyre et al., 2022). Existing research supports the long-term and regular use of acetaminophen to have an impact on blood pressure. However, perhaps the most important is whether these BP effects translate to increased cardiovascular risk (Smith and Cooper-DeHoff, 2022). There is not enough high-quality data to evaluate the cardiovascular risk of acetaminophen. In a large number of hypertensive patients, the use of acetaminophen is not associated with an increased risk of myocardial infarction or stroke (Fulton et al., 2015). Our study included a critically ill population diagnosed with gout and hypertension, with the primary endpoint set as in-hospital mortality, which better reflects the safety of acetaminophen in this population and suggests a reduction in in-hospital mortality. In addition, we further analyzed the effects of the dosage and duration of acetaminophen on in-hospital mortality and the occurrence of hypertension emergencies. Based on our research results, using a daily dosage range of 2–4 g acetaminophen and a short course of 3–5 days resulted in greater benefits than risks.

### 4.1 Study limitations

The current study has several key limitations. Firstly, because of its retrospective observational design, the results are subject to residual bias and unmeasured confounders despite propensity score matching and multivariable analyses. Secondly, the cause-effect relationship could not be established. Thirdly, due to the significant absence of serum uric acid values and the lack of information related to febuxostat, allopurinol, and benzbromarone, the impact of blood uric acid was not analyzed. Finally, the population we included in the study was predominantly white individuals, and further observations of other populations are needed in the future.

## 5 Conclusion

Acetaminophen use was associated with lower in-hospital mortality in critically ill patients with gout and hypertension. Prospective studies are needed to verify this retrospective finding.

## Data Availability

The original contributions presented in the study are included in the article/Supplementary Material, further inquiries can be directed to the corresponding authors.
