# Supplementary material for: Association between acetaminophen administration and outcomes in critically ill patients with gout and hypertension
Source: Front Pharmacol. 2024 Aug 12;15:1445975. doi: 10.3389/fphar.2024.1445975 (PMC11348437; doi:10.3389/fphar.2024.1445975)
Supplement: Supplementary file 1 [file Table1.DOCX]

Table S1. Missing statistics

|  | Miss.freq | Miss.percentage% |
| --- | --- | --- |
| BMI | 1145 | 46.77 |
| WBC, | 19 | 0.77 |
| Platelet, | 19 | 0.77 |
| ALT | 1246 | 50.89 |
| AST | 1239 | 50.61 |
| Serum creatinine | 10 | 0.40 |
| Serum glucose | 20 | 0.81 |
| Heart rate | 4 | 0.16 |
| SBP | 12 | 0.49 |
| DBP | 12 | 0.49 |
| Respiratory rate | 5 | 0.20 |
| Temperature(℃) | 92 | 3.75 |
| Spo2 | 4 | 0.16 |

Table S2. Baseline characteristics of entire cohort

| Variables | Overall (n = 2448) | No acetaminophen  (n = 1834) | Acetaminophen (n = 614) | P-value |
| --- | --- | --- | --- | --- |
| Demographics |  |  |  |  |
| Age, year | 73.1 ± 11.8 | 73.5 ± 12.1 | 71.9 ± 10.6 | 0.006 |
| Gender, Male, n (%) | 1817 (74.2) | 1326 (72.3) | 491 (80) | < 0.001 |
| Race, white, n (%) | 1688 (69.0) | 1275 (69.5) | 413 (67.3) | 0.296 |
| BMI, kg/m2 | 30.9 ± 7.6 | 30.9 ± 7.8 | 31.1 ± 7.1 | 0.639 |
| Laboratory Examination |  |  |  |  |
| WBC, K/µl | 10.7 (8.0, 14.3) | 10.1 (7.6, 13.6) | 12.6 (9.6, 15.9) | < 0.001 |
| Platelet, K/µL | 178.0 (135.5, 233.1) | 185.1 (141.4, 242.0) | 156.3 (126.3, 204.2) | < 0.001 |
| ALT, U/L | 24.0 (15.5, 48.5) | 25.0 (16.0, 49.0) | 23.8 (15.0, 46.4) | 0.147 |
| AST, U/L | 33.0 (21.0, 60.5) | 32.5 (21.0, 60.0) | 35.0 (22.0, 61.9) | 0.153 |
| Serum creatinine, mg/dL | 1.4 (1.0, 2.1) | 1.4 (1.0, 2.3) | 1.2 (0.9, 1.7) | < 0.001 |
| Glucose, mg/dL | 129.6 (108.5, 160.0) | 130.5 (108.5, 162.9) | 128.0 (109.0, 152.5) | 0.199 |
| Concomitant Medication |  |  |  |  |
| βblocker, n (%) | 2000 (81.7) | 1443 (78.7) | 557 (90.7) | < 0.001 |
| ACEI, n (%) | 728 (29.7) | 561 (30.6) | 167 (27.2) | 0.112 |
| ARB, n (%) | 318 (13.0) | 242 (13.2) | 76 (12.4) | 0.602 |
| CCB, n (%) | 710 (29.0) | 488 (26.6) | 222 (36.2) | < 0.001 |
| Diuretics, n (%) | 1719 (70.2) | 1211 (66) | 508 (82.7) | < 0.001 |
| Colchicine, n (%) | 392 (16.0) | 317 (17.3) | 75 (12.2) | 0.003 |
| NSAIDs, n (%) | 113 ( 4.6) | 84 (4.6) | 29 (4.7) | 0.884 |
| Glucocorticoids, n (%) | 380 (15.5) | 295 (16.1) | 85 (13.8) | 0.184 |
| Antibiotic | 1624 (66.3) | 1075 (58.6) | 549 (89.4) | < 0.001 |
| Vasoactive drugs | 911 (37.2) | 513 (28) | 398 (64.8) | < 0.001 |
| Comorbidities |  |  |  |  |
| Myocardial infarct, n (%) | 595 (24.3) | 441 (24) | 154 (25.1) | 0.605 |
| Congestive heart failure, n (%) | 1088 (44.4) | 862 (47) | 226 (36.8) | < 0.001 |
| Peripheral vascular disease, n (%) | 419 (17.1) | 313 (17.1) | 106 (17.3) | 0.911 |
| Cerebrovascular disease, n (%) | 375 (15.3) | 282 (15.4) | 93 (15.1) | 0.891 |
| Chronic pulmonary disease, n (%) | 651 (26.6) | 500 (27.3) | 151 (24.6) | 0.195 |
| Rheumatic disease, n (%) | 104 ( 4.2) | 82 (4.5) | 22 (3.6) | 0.345 |
| Peptic ulcer disease, n (%) | 73 ( 3.0) | 64 (3.5) | 9 (1.5) | 0.011 |
| Diabetes, n (%) | 1032 (42.2) | 799 (43.6) | 233 (37.9) | 0.015 |
| Renal disease, n (%) | 1217 (49.7) | 973 (53.1) | 244 (39.7) | < 0.001 |
| Liver disease, n (%) | 212 ( 8.7) | 175 (9.5) | 37 (6) | 0.007 |
| Vital signs |  |  |  |  |
| heart_rate | 81.6 ± 14.7 | 81.4 ± 15.2 | 82.4 ± 13.4 | 0.151 |
| SBP, mmHg | 119.9 ± 16.5 | 121.3 ± 17.1 | 116.0 ± 13.9 | < 0.001 |
| DBP, mmHg | 61.6 ± 11.0 | 62.4 ± 11.3 | 59.2 ± 9.6 | < 0.001 |
| Respiratory rate | 19.2 ± 3.5 | 19.3 ± 3.6 | 18.7 ± 3.1 | < 0.001 |
| Temperature(℃) | 36.8 ± 0.5 | 36.7 ± 0.5 | 36.8 ± 0.5 | < 0.001 |
| Spo2 | 96.7 ± 2.2 | 96.6 ± 2.2 | 97.2 ± 1.9 | < 0.001 |
| Scoring systems |  |  |  |  |
| SOFA | 5.0 (3.0, 7.0) | 4.0 (2.0, 6.0) | 6.0 (4.0, 8.0) | < 0.001 |
| Events |  |  |  |  |
| LOS hospital, days | 7.6 (4.8, 11.9) | 7.0 (4.3, 11.4) | 8.7 (6.1, 13.9) | < 0.001 |
| In-hospital mortality, n (%) | 215 ( 8.8) | 160 (8.7) | 55 (9) | 0.86 |
| 30-day mortality, n (%) | 295 (12.1) | 229 (12.5) | 66 (10.7) | 0.252 |
| 60-day mortality, (%) | 396 (16.2) | 312 (17) | 84 (13.7) | 0.052 |

Table S3. Associations between acetaminophen use and the outcome in the crude analysis, multivariable analysis, and propensity-score analyses.

| Analysis | In-hospital mortality(%) | P-value |
| --- | --- | --- |
| No.of events/no.of patients at risk(%) |  |  |
| No APAP use | 160/1834(8.7) |  |
| APAP use | 55/614(9) |  |
| Crude analysis-hazard ratio (95%CI) | 0.73 (0.54~0.99) | 0.044 |
| Multivariable analysis-hazard ratio (95%Cl) | 0.64 (0.45~0.91) | 0.014 |
| The inverse probability of treatment weighting(IPTW) | 0.67 (0.5~0.91) | 0.048 |
| With matching | 0.67 (0.46~0.98) | 0.041 |
| Adjusted for propensity score | 0.75 (0.53~1.05) | 0.092 |

**Table S4.** **Multivariable Cox regression analysis for LOS hospital**

| **Categories** | **Model I** |  | **Model II** |  | **Model III** |  |
| --- | --- | --- | --- | --- | --- | --- |
|  | **β** (95%CI) | P-value | **β** (95%CI) | P-value | **β** (95%CI) | P-value |
| **second outcome** |  |  |  |  |  |  |
| **LOS hospital, days** |  |  |  |  |  |  |
| Entire cohort (APAP) | 2.78(2~3.56) | <0.001 | 2.72(1.94~3.5) | <0.001 | 1.37(0.57~2.16) | 0.001 |
| PSM cohort (APAP) | 1.37(0.21~2.54) | 0.021 | 1.34(0.18~2.5) | 0.024 | 1.41(0.32~2.49) | 0.011 |

Model I: did not adjust any variables

Model II: adjusted for age, gender, race, BMI

Model III: adjusted for model II covariates, βblocker, ACEI, ARB, CCB, diuretics, colchicine, vasoactive drugs, congestive heart failure, chronic pulmonary disease, SBP, DBP, respiratory rate, WBC, serum creatinine, SOFA.
